# Functional MRI Studies in Friedreich's Ataxia: A Systematic Review

**DOI:** 10.3389/fneur.2021.802496

**Published:** 2022-03-10

**Authors:** Marinela Vavla, Filippo Arrigoni, Denis Peruzzo, Domenico Montanaro, Francesca Frijia, Silvia Pizzighello, Alberto De Luca, Emma Della Libera, Federica Tessarotto, Paola Guerra, Ian H. Harding, Andrea Martinuzzi

**Affiliations:** ^1^Department of Neurorehabilitation, Pieve di Soligo, Scientific Institute, IRCCS E. Medea, Pieve di Soligo, Italy; ^2^Neuroimaging Lab, Scientific Institute, IRCCS Eugenio Medea, Bosisio Parini, Italy; ^3^U.O.C. Risonanza Magnetica Specialistica e Neuroradiologia, Fondazione CNR/Regione Toscana G. Monasterio, Pisa, Italy; ^4^U.O.S.D. Servizio Autonomo di Risonanza Magnetica, Dipartimento Clinico di Neuroscienze dell'Età Evolutiva - IRCCS Fondazione Stella Maris - Pisa, Italy; ^5^U.O.C. Bioingegneria e Ing. Clinica, Fondazione Toscana Gabriele Monasterio, Pisa, Italy; ^6^Department of Neurology, UMC Utrecht Brain Center, UMC Utrecht, Utrecht, Netherlands; ^7^CIBIO, University of Trento, Trento, Italy; ^8^Department of Neuroscience, Central Clinical School, Monash University, Melbourne, VIC, Australia

**Keywords:** Friedreich's ataxia, functional magnetic resonance imaging, systematic review, clinical study, study design, fMRI protocol

## Abstract

Friedreich's ataxia (FRDA) is an inherited neurodegenerative movement disorder with early onset, widespread cerebral and cerebellar pathology, and no cure still available. Functional MRI (fMRI) studies, although currently limited in number, have provided a better understanding of brain changes in people with FRDA. This systematic review aimed to provide a critical overview of the findings and methodologies of all fMRI studies conducted in genetically confirmed FRDA so far, and to offer recommendations for future study designs. About 12 cross-sectional and longitudinal fMRI studies, included 198 FRDA children and young adult patients and, 205 healthy controls (HCs), according to the inclusion criteria. Details regarding GAA triplet expansion and demographic and clinical severity measures were widely reported. fMRI designs included motor and cognitive task paradigms, and resting-state studies, with widespread changes in functionally activated areas and extensive variability in study methodologies. These studies highlight a mixed picture of both hypoactivation and hyperactivation in different cerebral and cerebellar brain regions depending on fMRI design and cohort characteristics. Functional changes often correlate with clinical variables. In aggregate, the findings provide support for cerebro-cerebellar loop damage and the compensatory mechanism hypothesis. Current literature indicates that fMRI is a valuable tool for gaining *in vivo* insights into FRDA pathology, but addressing that its limitations would be a key to improving the design, interpretation, and generalizability of studies in the future.

## Introduction

Friedreich's ataxia (FRDA) is an inherited neurodegenerative movement disorder that affects the cerebellum and cerebellar pathways to various degrees. Patients with FRDA present with equilibrium impairment, gait ataxia, dysmetria, nystagmus, oculomotor disturbances, tremor, dysarthria, and non-neurological impairments, including cardiomyopathy (1). This condition is transmitted in a recessive autosomal mode, most commonly due to biallelic triplet repeat expansions in the *FXN* gene (2), which leads to impaired expression of the protein frataxin (3). Reduced transcription of frataxin leads to impaired mitochondrial function and subsequent cell death in vulnerable tissues. In the brain, the principal sites of neuropathology in FRDA are the dorsal root ganglia in the spinal cord, cerebellum, and cerebellar tracts although there is increasing evidence of more widespread cerebral pathology and dysfunction, and extensive white matter damage (4).

To date, there is no cure and only early evidence of efficacious disease-modifying treatments for FRDA. However, there is an ever-growing body of research investigating the disease at multiple levels of biology (i.e., from genes to systems), alongside an increasing range of therapeutic trials.

One influential and growing body of research has been *in vivo* studies of patients with FRDA using MRI techniques, which have highlighted insights into brain structure and function in FRDA cohorts compared to healthy control (HC) groups.

Structural MRI findings in FRDA have shed light on atrophy affecting infratentorial and supratentorial structures, with reports of regional reductions in cerebral, cerebellar, and brainstem volume (5–10) and additional reports of decreased size of the dentate nuclei (DN) (11–13). In particular, the pattern of cerebellar atrophy indicates no overall but discrete lobular distribution of cortical atrophy (14). These findings are supported by the diffusion tensor imaging (DTI) studies that have documented widespread abnormalities in cerebro-cerebellar and corticospinal white matter (5, 15–24).

Several functional MRI (fMRI) studies have also been conducted in patients with FRDA from research groups all over the globe, particularly in the past decade. These studies have explored the activation of different central nervous system (CNS) areas by means of both motor and cognitive tasks (language, memory, and behavioral) in patients compared to HC groups. Relative to structural changes, which pinpoint the areas of cell loss, functional studies allow the assessment of changes in dynamic activation and metabolism in (at least partially) intact neuronal populations. These studies provide critical insights into pre-apoptotic cellular dysfunction and large-scale *cerebral*–*cerebellar* circuitry damage in FRDA, including evidence of downstream diaschisis or compensation. These findings could be important as landmarks for future study designs, provide more insights into the pattern and time course of neurodegeneration, and may perhaps even contribute to the development and monitoring of novel strategies for cerebellar rewiring and therapeutic monitoring.

In our systematic review of fMRI studies conducted in FRDA so far, we aimed, as a first objective, to perform a critical appraisal of the findings regarding changes in the cerebral and cerebellar circuitry underlying motor and cognitive tasks, and “intrinsic” brain connectivity measured during the resting-state fMRI (RS-fMRI), in people with FRDA. Our second aim was to provide recommendations regarding the use of fMRI in future study design, biomarker development, and therapeutic or intervention proposals in this disease.

## Methods

This systematic review was written in accordance with the PRISMA statement (25).

### Search Strategy

Two authors (MV and FF) independently performed a comprehensive literature search using the PubMed database, to find relevant studies for the systematic review on brain fMRI in FRDA. The search included a series of eligibility criteria (see section Eligibility Criteria). The search string included the following keywords “FRIEDREICH('S) ATAXIA,” “FUNCTIONAL MAGNETIC RESONANCE,” “fMRI,” and “BRAIN.” The search was performed within the time range from January 1, 1996 to October 31, 2020.

### Eligibility Criteria

The eligibility criteria for the studies considered were original research (either cross-sectional or longitudinal studies), on genetically confirmed patients with FRDA (triplet expansion and mutations in *FXN* gene), brain fMRI study, publication period starting from January 1, 1996 and papers in English language.

The exclusion criteria were case reports, review papers, conference proceedings, thesis, comments, editorials or letters, and other diagnosis and other neuroimaging technique studies.

### Data Collection Process

After the final selection of the papers eligible for a systematic review, elements were extracted to provide the following information for each paper: reference, year of publication, journal, first author's country and affiliation, category of clinical research and experimental design, FRDA and HC group size, gender ratio in FRDA and HC groups, disease duration and age at onset in the FRDA group, short-allele GAA (GAA1) length, long-allele GAA (GAA2) length, age of participants at the fMRI scan, education (years of schooling) in FRDA and HC groups, disease severity measures, hand dominance, cognitive and psychological measures used and findings, other clinical measures, and concurrent pharmacological treatments.

Regarding the fMRI technique for each study, we collected the following data on: field strength, head coil channels, acquisition parameters [repetition time (TR), echo time (TE), flip angle, slice orientation, amount and thickness, matrix size, in plane resolution, field of view (FOV)], fMRI post-processing software, anatomic references, any fMRI task applied (motor and nonmotor task paradigms), task-related devices, if RS-fMRI was collected, region of interest (ROIs) analysis used for the studies of the blood oxigenation level dependent (BOLD) signal time series, correlations with clinical features, fMRI findings divided into supratentorial and infratentorial and intragroup and intergroup data, novelties and comments about the analysis approach.

### Summary Measures and Synthesis of Results

This study was intended as a systematic review, and no quantitative analysis was performed.

All the variables considered (in section Data Collection Process) were collected in a unique spreadsheet and were simultaneously checked by authors independently (MV, FA, DP, DM, FF, and SP). Subsequently, the papers were read and analyzed according to the following main points: demographic and clinical characteristics of the patients, fMRI paradigms of every study, general results of fMRI studies grouped as motor or non-motor, cognitive and neuropsychological results, and finally technical aspects of the fMRI scans and statistical analysis applied in each study.

## Results

### Literature Search

**Figure 1:**
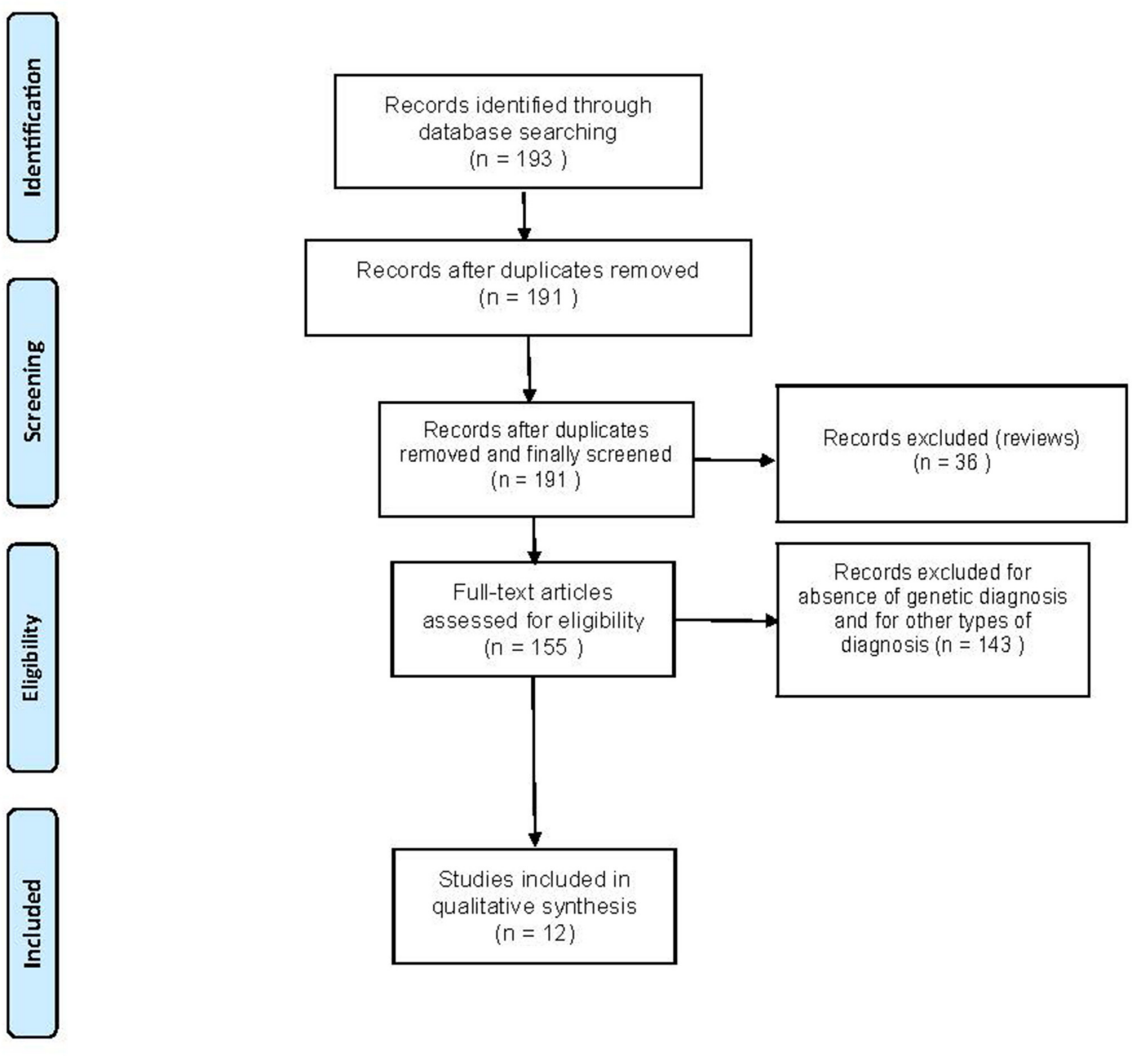
Flowchart of the search for eligible studies on the functional MRI (fMRI) findings in Friedreich's Ataxia (FRDA).

Overall, 193 records were identified through a comprehensive computer-based search of the PubMed database. In addition, we checked the reference list of selected articles for additional relevant papers. About 181 records were excluded because they were not relevant to the systematic review as they consisted of the studies focusing on susceptibility-weighted imaging (SWI), iron imaging, diffusion tensor imaging (DTI), volumetric MRI, magnetic resonance spectroscopy (MRS), positron emission tomography (PET), single-photon emission CT (SPECT), diagnoses other than FRDA, video neuroimaging, cardiac function, did not genetically confirm FRDA diagnoses, were written in languages other than English, and review articles. After exclusions, 12 articles satisfied the inclusion criteria and underwent qualitative synthesis control. Figure 1 presents the results of the literature search.

Table 1 contains the demographic and clinical data, and Table 2 contains fMRI paradigms and technical characteristics of the studies.

**Table 1:** Demographic and clinical data.

**Reference (country)**	**Journal (year)**	**Category of clinical research**	**Experimental design**	**FRDA sample size (F/M) analyzed**	**HCs sample size (F/M)**	**FRDA Age at fMRI (mean ±SD, or range in yrs)**	**HCs age at fMRI (mean ±SD, or range in yrs)**	**FRDA DD (mean ±SD, range in yrs)**	**FRDA AAO mean ±SD (range), yrs**	**GAA1**	**GAA2**	**Severity measure scale: mean ±SD (range of values)**	**Treatment**
Mantovan M. C. (Italy) (26)	European Journal of Neurology (2006)	Original research	Cross-sectional FRDA vs. HC	13 (7/6)	4 (n.r.)	23.7 ± 9.7	n.r.	13.7 ± 9.3 (range 3–29)	n.r.	n.r.	n.r.	n.r. (able to perform finger tapping and no dysartria)	n.r.
Ginestroni A. (Italy) (27)	Human Brain Mapping (2012)	Original research	Cross-sectional FRDA vs. HC	Task 1: 11 (6/5); Task 2: 7 (3/4)	Task 1: 13 (6/7); Task 2: 9 (3/6)	Task 1: 34.3 ± 8.5; Task 2: 38.6 ± 7	Task 1: 31.9 ± 10.7; Task 2: 34.4 ± 6.5	Task 1: 11.4 ± 4.3; Task 2: 12.6 ± 5.2	n.r.	Task 1: 508 ± 236; Task 2: 463 ± 287	n.r.	IACRS: Task 1 21.6 ± 7.4; Task 2 21.5 ± 7.1	n.r.
Akhalaghi H. (Australia) (28)	Brain Research (2012)	Original Resaerch	Cross-sectional FRDA vs. HC	11 (6/5)	13 (8/5)	35.1 ± 9.6	33.1 ± 7.8	15 ± 6	19 ± 6	649 ± 205	1,007 ± 107	FARS: 88 ± 15 (63–113)	n.r.
Georgiou-Karistianis N. (Australia) (29)	Brain and Cognition (2012)	Original research	Cross-sectional FRDA vs. HC	13 (7/6)	14 (5/9)	35.5 ± 9.4	33.7 ± 7.9	15.7 ± 5.8	20 ± 6.5	608 ± 243	n.r.	FARS: 86.5 ± 15.7	n.r.
Stefanescu M. R. (Germany) (12)	Brain (2015)	Original research	Cross-sectional FRDA vs. HC	12 (7/5)	12 (7/5)	39.08 ± 12.87 (21–55)	40 ± 13.2 (22–59)	19 ± 9.7 (3–32)	21 ± 6.6 (11–31)	n.r.	n.r.	SARA: 19.6 ± 6.37 (12–31.5)	n.r.
Dogan I. (Germany) (30)	Annals of Clinical and Translational Neurology (2016)	Original research	Cross-sectional FRDA vs. HC	15 (7/8)	15 (7/8)	37.73 ± 13.57	39.2 ± 12.62	18.33 ± 9.09	19.4 ± 7.98	469.6 ± 229.7	771.93 ± 252.09	SARA 20.1 ± 7.34, INAS 4.73 ± 2.37, SCAFI	Reported
Harding I.H. (Australia) (13)	Human Brain mapping (2016)	Original research	Cross-sectional FRDA vs. HC	29 (13/16)	34 (17/17)	30.0 (18.2–56.3)	33.6 (18.8–62.1)	15.4 ± 7.81	19.4 ± 9.01	546 ± 231	860 ± 251	FARS: 88 (19–119)	Reported
Harding I.H. (Australia) (31)	Movement Disorders (2017)	Original research	Cross-sectional FRDA vs. HC	25 (10/15)	33 (17/16)	34.9 ± 12.1	36.9 ± 13.1	14.9 ± 6.6	19.8 ± 9.3	553 ± 232	844 ± 221	FARS: 77.4 ± 23.7	n.r.
Cocozza S. (Italy) (32)	Annals of Clinical and Translational neurology (2018)	Original research	Cross-sectional FRDA vs. HC	24 (9/15)	24 (9/15)	31.3 ± 15	30.7 ± 15.5	n.r.	n.r.	677 ± 282.8	906.3 ± 310.4	SARA: 18.7 ± 7.2	n.r.
Vavla M. (Italy) (20)	Frontiers in Neurology (2018)	Original research	Cross-sectional FRDA vs. HC	14 (12/2)	15 (10/5)	27.6 ± 11.1 (12.1–50.5)	27.9 ± 9.8 (15.9–45.7)	16.33 ± 8.82 (3–32)	10.62 ± 4.58 (4–20)	671.24 ± 210.5 (170–946)	812.6 ± 225.04 (350–1,230)	SARA 21.38 ± 7.76 (8–32), ICARS 52.95 ± 18.53 (22–84), FARSne 62.25 ± 19.37 (31.33–92.5)	n.r.
Shishegar R. (Australia) (33)	Cerebellum (2020)	Original research	Longitudinal FRDA vs. HC (24 months)	21 (6/15)	28 (14/14)	35.23 ± 2.65	38.41 ± 2.53	13.83 ± 1.49	21.24 ± 2.06	476.14 ±4 0.09	830.81 ± 57.21	FARS: baseline 71.21 ± 6.48, f-up 77.94 ± 6.47	n.r.
Vavla M. (Italy) (34)	Frontiers in Neuroscience (2020)	Original research	Open Label Phase II, Longitudinal (4 time- points)	10 (4/6)	0	16.6 ± 4.55 (11–26)	n.a.	At baseline: 8.6 ± 4.45 (2–14)	8 ± 2.94 (4–12)	683.8 ± 131.43 (460–862)	965.4 ± 145.1 (750–1,166)	SARA Baseline: 17.55 ± 5.88 (7.5–25)	IFNy

*FRDA, Friedreich's ataxia; HCs, healthy controls; F, female; M, male; SD, standard deviation; DD, disease duration; AAO, age at onset; fMRI, functional MRI; yrs, years; GAA1, short allele GAA; GAA2, long allele GAA; IACRS, inherited ataxia clinical rating scale; FARS, Friedreich's ataxia rating scale; SARA, scale for the assessment and rating of ataxias; INAS, inventory of non-ataxia symptoms; SCAFI, spinocerebellar ataxia functional index; ICARS, International Cooperative Ataxia rating scale; n.r., not reported; IFNγ, interferon gamma*.

**Table 2:** fMRI paradigm.

**Reference**	**fMRI (Field strength/Vendor)**	**Channel head coil**	**Handedness (R, L)**	**Motor task**	**Resting-state**	**NPS/Language Task-fMRI**	**Device**	**fMRI post-processing software**	**Anatomic References**	**ROI regions for BOLD-signal time-series study**	**Other neuroimaging techniques**
Mantovan M. C. (Italy) (26)	1.5T (GE HC)	n.r.	R	Block design: Box-Car (30 s), 2 on-3 off, 2 Hz finger-tapping of dominant hand	n.a.	No	None	SPM (version n.r.)	Talairach atlas	Transverse oblique orientation for cerebellum, primary SM cortex.	MRI atrophy index, SPECT
Ginestroni A. (Italy) (27)	1.5T (Philips Intera)	n.r.	R FRDA, R HCs, (Edinburgh Test)	1: Hand-tapping task; 2: writing of “8” task	n.a.	n.a.	Right hand glove in-house developed MRI for movements grades evaluation device. Video recorded.	FSL, FMRIB's Soft Libr.	Talairach standard space	Only in discussion. No ROI selections. Not discussed signal intensity	DTI, VBM
Akhalaghi H. (Australia) (28)	3.0T (Siemens TRIOTim)	8 ch	R	(1) visually cued, regular timed single finger tapping; (2) visually cued, irregular timed single finger tapping; (3) visually cued, regular timed random multi-finger tapping; (4) un-cued self-paced regular single finger tapping	n.a.	n.a.	R hand glove with movement sensors	fMRI FEAT vers 5.98 (FSL, FMRIB's Soft Libr)	MNI atlas space	(1) L M1 (activated); (2) L BG (MNI on Putamen); (3) L SMA + R iPL = Fronto-Parietal loop (activated); (4) R cerebellum lobules (V+VI) (activated).	VBM (brain, cerebellum)
Georgiou-Karistianis N. (Australia) (29)	3.0T (Siemens TRIOTim)	8 ch	n.r.	n.a.	Functional Connectivity (n.r. in the methodology)	Simon effect task	Screen: not described	fMRI FEAT vs. 5.98 (FSL, FMRIB's Soft Libr)	MNI atlas space	n.r.	n.r.
Stefanescu M. R. (Germany) (12)	7.0T (Siemens HC)	32 ch	10 R, 2 L (FRDA and HCs)	R lower arm rested on the thigh, opening and closing movements of R fist at rate at 1.66Hz	n.a.	n.a.	Pace-movements with tone using plastic tubes and small sponge ear plugs and background color changing between white and red in the MRI bore using a projection screen. MR- compatible Glove for monitoring amplitude and frequency in movements off-line.	SPM8	MNI atlas space	M1, Cerebellar cortex, DN	VBM (cerebellar cortex, DN), SWI (DN)
Dogan I. (Australia) (30)	3.0T (Siemens TRIOTim)	n.r.	12R FRDA, 12R HCs	n.a.	Functional connectivity (reported in methods)	Phonemic task. Semantic task. Overt speech. Covert speech.	Projected: not reported devise	SPM8 fMRI and VBM. FSL, FMRIB's Soft Libr DTI	MNI atlas space	BA44, BA45, insula, ACC, M1 (4a/4p), cerebellar lobules VI, VIIa, VIIb, Crus I/II.	DTI, VBM
Harding I.H. (Australia) (13)	3.0T (Siemens Skyra)	32 ch	FRDA 33R/1L; HCs 27R/1L/1 no clear preference	n.a.	Task-related functional connectivity using generalized psychophysiological al interaction analyses	N-Back: 0-back, 2- back.	Stimuli were presented using E-prime software (Psychological Software Tools, Pittsburgh), projected centrally onto a translucent screen and viewed through a head coil mounted mirror. Responses were collected *via* LumitouchTM fMRI optical response keypads	SPM8	MNI atlas space	For functional connectivity seed ROI: R lobule VI.	n.r.
Harding I.H. (Australia) (31)	3.0T (Siemens Skyra)	32 ch	FRDA 24R/1L; HCs 33R	Speeded tapping, paced tapping; self-paced motor task, multifinger accuracy	n.a.	n.a.	E-prime (Psychological Software Tools, Pittsburgh, Pennsylvania). MRIcompatible, R-handed glove with movement sensors (Fifth Dimension Technology, www.5DT.com) to record the kinematics of the finger movements.	SPM12	Atlas of intrinsic functional human	n.r.	n.a.
Cocozza S. (Italy) (32)	3.0T (Siemens TRIO)	n.r.	n.r.	n.a.	RS-fMRI	Patients were asked to relax with eyes closed, without falling asleep.	n.a.	SPM8	MNI atlas space	39 functional seeds: Frontal/Temporal/Parietal/Occipital lobes, Deep GM.	n.r.
Vavla M. (Italy) (20)	3.0T (Philips Achieva)	32 ch	FRDA 14R; HC 15 R	Standard block design finger tapping task for both hands	n.a.	n.a.	Subjects were asked to press the buttons of an MRI-compatible response-device, paced according to the screen commands provided with a regular pattern. A drawing of the R or L hand with a caption projected on MR compatible goggles	SPM12	MNI atlas space	ROI based GLM correlation analysis. 8 ROIs related to the movement of the R hand, 7 ROIs related to the movement of the L hand.	VBM, DTI
Shishegar R. (Australia) (33)	3.0T (Siemens Skyra)	32 ch	n.r.	n.a.	n.a.	“0-back” and “2-back” N-Back Working Memory Task, with letters.	Stimuli were presented using E-prime software (Psychological Software Tools, Pittsburgh), projected centrally onto a translucent screen and viewed through a head coil mounted mirror. Responses were collected *via* LumitouchTM fMRI optical response keypads	SPM12	MNI atlas space for Cerebrum. SUIT space for cerebellum	Group-level inferences from a priori mask from atlas of the VAN: precental gyrus, superior frontal gyrus, inferior frontal gyrus, pars opercularis, SMA, supramarginal gyrus, middle temporal gyrus, midcingulate area, medial frontal gyrus, insula and precuneus	VBM
Vavla M. (Italy) (34, 35)	3.0T (Philips Achieva)	32 ch	FRDA: 10R	Standard block design finger tapping task for both hands	RS-fMRI	n.a.	Subjects were asked to press the buttons of an MRI-compatible response-device, paced according to the screen commands provided with a regular pattern. A drawing of the R or L hand with a caption projected on MR compatible goggles	FSL, ANTs, SPM	MNI atlas space	motor task fMRI: M1; cerebellum; RS-fMRI: DMN, Hippocampus, Bilateral Fronto-Parietal, Bilateral Fronto-Temporal, Visual and Motor Network	DTI

*Right, R; L, Left; FRDA, Friedreich's ataxia; HCs, healthy controls; NPS, neuropsychological, Fmri, functional MRI; SPECT, single-photon emission CT; DTI, diffusion tensor imaging; VBM, voxel-based Morphometry; RS-fMRI, resting-state fMRI; SWI, susceptibility-weighted images; MNI, Montreal Neurological Institute; GLM, general linear model; DMN, default mode network; ROI, region of interest; BOLD, blood oxygenation level dependent; SPM, Statistical parametric mapping; M1, primary motor; SMA, sensorimotor area; BG, Basal Ganglia; iPL, inferior parietal lobe; DN, dentate nuclei; ACC, anterior cingulated cortex; BA, Brodmann area; VAN, ventral attention network; GM, gray matter; n.a, not applied; n.r., not reported*.

### Demographic and Clinical Characteristics

About 12 papers analyzed for this systematic review are original research articles that were published in a peer-review journal over an ~14-year period from 2006 to 2020 (Table 1).

These studies have been conducted in Europe (*n* = 5 Italy and *n* = 2 Germany) and in Australia (*n* = 5).

The main demographic and clinical characteristics analyzed in the papers are detailed in Table 2:

- Two are cross-sectional with a FRDA group and a HC group. One study performed imaging in different types of patients with ataxia, including FRDA, SCA3, and SCA6, analyzing the groups separately and comparing with HC groups (12).
- The fMRI studies have analyzed the data from 198 patients with FRDA and 205 HCs, with sample size ranging from 10 to 29 people with FRDA and 4–34 HCs. Gender was reported in almost all the studies, with the numbers that reflected a quite similar gender representation.
- Age-related variables were variably reported in all the studies.
- Several studies reported the schooling variable in both groups as years of education. The FRDA group schooling years varied with mean values of 9.7–15.5 years, and the HC groups with mean values of 10.9–17.5 years.
- Diagnoses were based on Harding's clinical criteria confirmed by molecular tests on genomic DNA from peripheral blood (2, 36). GAA triplet size was variably reported in all studies.
- Clinical severity measures were reported in all the studies but one. Different standardized ataxia scales were used across the studies.
- Treatment of the FRDA group was reported only in two studies, mentioned as chronic treatment (13, 30) or an ongoing open-label phase II study (34, 35).

### fMRI Paradigms

Functional MRI paradigms are reported in Table 2. About 7 of the 12 studies used motor tasks during the fMRI acquisition. The remaining studies implemented non-motor tasks assessing response inhibition (29), language (30), and working memory tasks (13, 33). One study presented a pure RS-fMRI study without any in-scan task (32). Vavla et al. (34) reported both a motor task and the RS-fMRI data.

A few studies on the implementation of motor task fMRI provided information regarding the in-scan device used for performance tracking. Conversely, Mantovan et al. (26) designed it without any device at all. The tapping tasks were performed with the glove devices that track finger movements (12, 27, 28) or a button press device (20, 34).

Screen projections were used by most studies to present in-scanner instructions to the participants for the cognitive and motor tasks (12, 13, 20, 29–31, 34).

The studies included in this systematic review were performed using scanners with different field strengths: 1 with an ultra-high field MRI scanner (7 Tesla); 9 with 3 Tesla MRI scanners, and 2 with a 1.5 Tesla scanner. The sequence used for the evaluation of the BOLD contrast was gradient-recalled echo type echo planar images (GRE-EPI) in all studies.

In 8 of the selected papers, the data were analyzed using Statistical Parametric Mapping (SPM Functional Imaging Laboratory, University College London, UK; www.fil.ion.ucl.ac.uk/spm/), sometimes exploiting the available extension toolboxes, while the remaining 3 studies rely on the FSL suite (FMRIB's Software Library, www.fmrib.ox.ac.uk/fsl). One study used both SPM and FSL toolboxes.

Only four studies consisted of unimodal fMRI protocols, while most of the others included a multimodal protocol, providing brain morphometry data (seven studies), DTI data (four studies), or SPECT imaging (one study) in addition to the fMRI technique.

### Analysis of fMRI Data

#### Subject-Level Analysis

All task-based studies (10 of 12 selected papers) performed a standard preprocessing pipeline and used a linear model to identify the activation maps (i.e., at a voxel level) for each subject recruited in the study.

Conversely, the two RS-fMRI studies used different approaches. Cocozza et al. (32) computed for each subject the functional connectivity maps related to 39 *a priori* selected ROIs. In Vavla et al. (34), an Independent Component Analysis was performed at a group level to firstly identify and select the common networks; subsequently, a dual regression analysis was performed to generate the subject-level spatial maps and time series for second-level analysis.

#### Group-Level Analysis

The selected manuscripts showed a more heterogeneous approach in the second-level analysis, i.e., when the subject activation maps are used for intergroup and intragroup analyses.

In general, a linear model (either fixed, random, or mixed effects) was used to fit covariates, group effects, and/or longitudinal effects to the functional summary statistics estimated at the first level (9 of 12 studies). Subsequently, statistical tests were performed on the model parameters and/or residuals to investigate significant differences. Alternatively, one study performed only a qualitative evaluation of the group differences by a visual inspection (26), while the two task fMRI studies exploited a two-stage Bayesian method (37) to perform intragroup and intergroup analyses (28, 29).

Activation map analyses were generally performed at a voxel level across the whole brain (at least for exploratory purposes) (27–29, 31), restricted to some ROIs that were identified either *a priori* (12, 30), data-driven (13, 20, 34), or a with a mixed approach (33). The ROI-based approach was usually exploited for detailed *post*-*hoc* analyses, where the activation values measured in the significant clusters/ROIs, the average time series, or the ROI-based connectivity measures were further investigated.

Different methods can be highlighted also in the correction for multiple comparisons. The most commonly used approach set the significance threshold to *p* < 0.05 corrected for multiple comparisons with the family wise error (FWE) approach performed at a cluster level (i.e., the significant threshold is computed on the cluster size). However, other approaches included correction for false discovery rate (20), bootstrapping methods (12), or the use of more conservative significant thresholds, such as *p* < 0.001 or *p* < 0.01 (12). Even mixed approaches were used in the same study for the different statistical tests (12, 30, 34).

### General Results

All the studies reported widespread changes in functionally activated areas in patients. The heterogeneity of reported changes and the differences among the study methodologies make it difficult to compose a summary of all the results. Therefore, we choose to describe the results for single studies assembled into two main categories: studies with motor and non-motor tasks (Supplementary Material S1).

**Figure 2:**
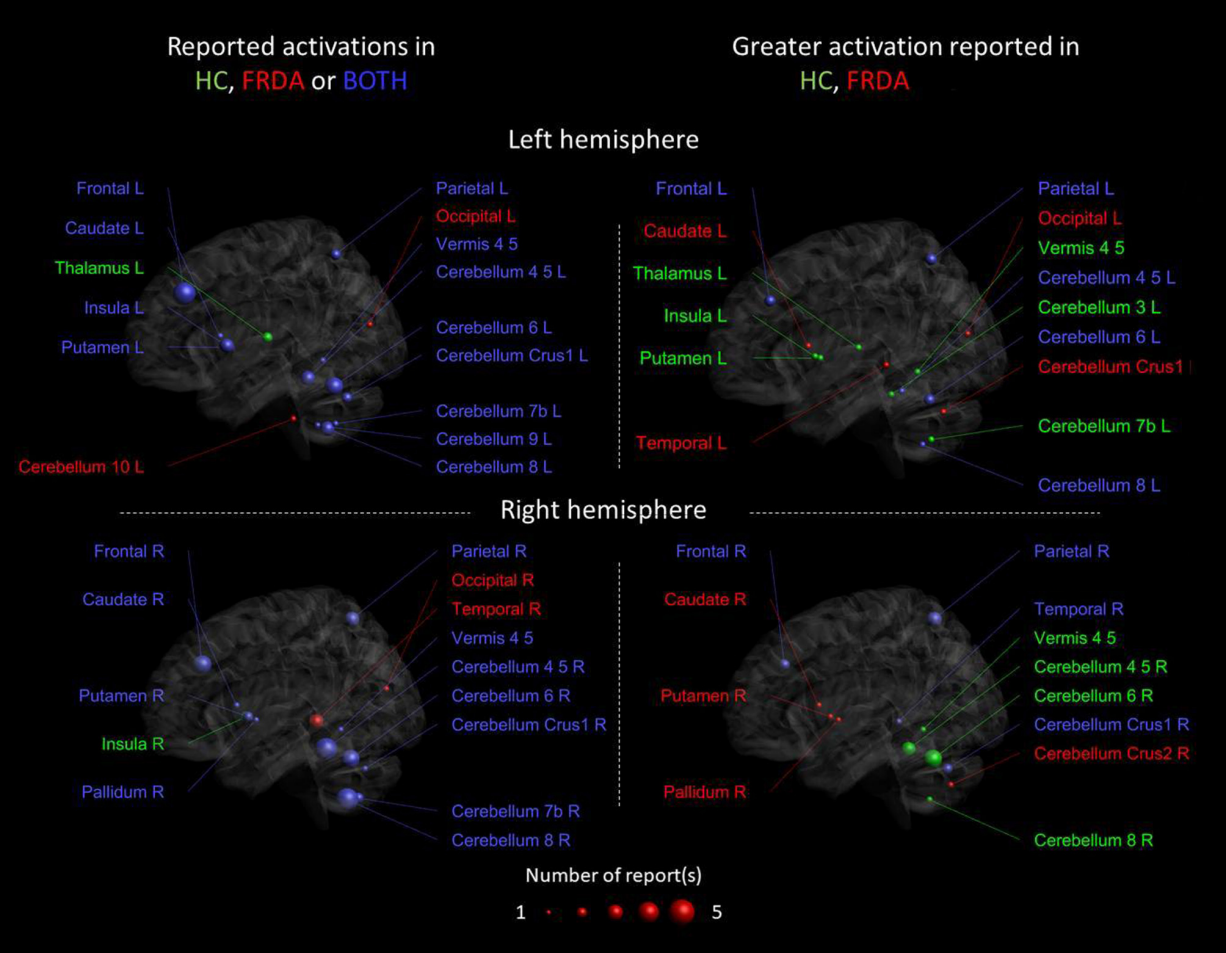
Cerebral and cerebellar areas reported as activated in the studies that performed a motor task fMRI. The colors of the nodes reflect activation in the respective groups as reported from each study: green [healthy controls (HCs)], red (FRDA), and blue (both groups). The panels on the left show the reported activated areas for each group or both groups, the panels on the right show the differential activation consistently reported when FRDA was compared to controls. The blue areas in the right panels are the areas in which the reports are conflicting. The size of the nodes reflects the number of papers reporting activation in a particular area, reported for each right and left hemisphere.

The motor fMRI studies are diverse in terms of fMRI paradigms, motor task modality, and results presentation. Nevertheless, considering that hand coordination was the common feature of all studies, we have summarized the results by illustrating the location and magnitude of activity across all studies (Figure 2). Overall, 36 cerebral and cerebellar areas were considered for the analysis, and these corresponded to the areas reported to be activated in the studies (Supplementary Table S2).

Figure 2 left panels show the areas activated during the motor task only in HC, only in FRDA or in both groups in the right and left hemisphere, respectively. Most areas were found activated in both groups, including the bilateral areas of the frontal and parietal lobe, the left and right putamen, and cerebellum lobules IV–VIII. The left thalamus and right insula were only activated in the HC group, whereas the bilateral occipital lobe, right temporal lobe, and left cerebellum lobule X only in the FRDA group. Altogether, these findings support the idea that both groups use similar areas to perform the motor tasks with similar activation patterns. Nevertheless, the regions reported activated only in HC or FRDA groups, which could indicate functional shifts in FRDA as an attempt for compensation. Nevertheless, the number of studies reporting activation in those areas is limited and deserves further investigation.

Figure 2 right panels show areas with an increased activation in HCs vs. FRDA, a greater activation in patients with FRDA vs. HCs (red), or both (contradictory results). The results suggest that there is a stronger activation in HCs in the vermis and the left thalamus/insula/putamen, although this was observed only in a study. Conversely, a stronger activation in FRDA was reported in the right caudate/pallidum/putamen, again in a single study. The most consistent finding across studies was the reduced activity in FRDA vs. HCs in right cerebellar lobules VI–VI. However, contradictory results were observed in many brain areas, including the bilateral frontal and parietal lobes, and the cerebellum. Overall, inconsistencies across the seven reviewed studies suggests that care is necessary when considering these results, and indicate that task design, cohort characteristics, or the test–retest reliability of findings may impact the generalizability of individual studies.

### Cognitive and Neuropsychological Results

Most of the studies selected for the review implemented out-of-scanner cognitive and psychological examinations in addition to in-scanner fMRI. Here, we report briefly only the results included in the selected papers without considering all the literature in FRDA. Of the 12 studies included in this review, only 3 did not include cognitive/psychological assessments (12, 27, 34).

The protocols used across the studies are overall very different and consisted of different tests used to assess similar functions (Supplementary Table S1), as well as a variety of tasks adopted as simple behavioral tasks (involving movements or visual activities) and others requiring more complex cognitive abilities (such as working memory or executive functioning).

Several studies included the assessment of cognitive ability, which was reported as normal in people with FRDA (20, 26, 30, 32) although one study reported decreased pre-morbid cognitive functioning in FRDA vs. HCs (29).

Language was explored in three studies, with normal language production (32), verbal fluency (26, 30), or semantic fluency (30).

Several components of attention, memory, and executive functioning were explored, and the studies were in agreement with general impairment in working memory, visuo-perceptual, and visuo-constructive functions. The psychological assessments showed pathological profiles in half of the patients (26). Dogan et al. (30) observed an impairment in the social abilities although this could be related to the impairment of working memory and executive functioning. Depression and anxiety were explored but no differences emerged when compared to the HC groups (13, 28–30).

## Discussion

The studies included in this systematic review represent encouraging attempts, by a few groups in a time period of 14 years, to explore the functional brain activity in FRDA with progressive improvements in fMRI techniques and with hypothesis-driven study designs, covering either motor or non-motor aspects in this condition. Most of these studies had a cross-sectional design, alongside a single longitudinal exploratory study and one clinical trial. All the studies presented a HC group well-matched to the FRDA cohort. No studies focusing on the spinal cord were retreived so far.

### Motor Task fMRI Studies

Studies implementing motor tasks (Figure 2) included children and adults and unimodal and multimodal MRI protocols, and data were acquired using MRI scanners with a magnetic field strength ranging from 1.5 to 7 Tesla. Most studies involved a comparison with a HC group and two involved a longitudinal evaluation including the first and only clinical trial with fMRI-based outcomes in FRDA.

Intergroup analyses showed the areas of hypoactivation and hyperactivation in FRDA in comparison with HCs in a widespread and variable pattern. These areas include widespread regions of the frontal, parietal, and temporal cortices, with particularly consistent involvement of the left precentral and bilateral postcentral gyri. Some areas were always reported as hypoactivated (left central opercular and left insular cortex) and others as only hyperactivated (left middle occipital, medial precuneus, and right fusiform gyrus), while more inconsistent results were found in frontal areas such as the sensorimotor area (SMA) and premotor area (PMA), which showed either bilateral hypoactivation or left hyperactivation results depending on the task and cohort. Similarly, subcortical areas showed either hypoactivation predominantly on the left (thalamus and putamen) or hyperactivation more prevalently on the right Basal Ganglia (BG) nuclei. Likewise, there appears to be an overlap of the cerebellar areas reported as hypoactivated and hyperactivated in FRDA in cerebellar lobules V, VI, VIII, and crus I/II. Interestingly, the ultra-high field MRI study registered hypoactivation in the DNs.

Several studies, but not all, reported correlations between brain activity and clinical variables. Taken together, clinical progression appears to be associated to reduced activation in areas such as SMA, parietal and temporal lobes, insula, cingulum, striatum, and cerebellum. Conversely, the results from the clinical trial showed reduced clinical severity scores concomitant with increased activity in the right precentral cortex for right and left hand motor task during the treatment period. The GAA1 triplet size inversely correlated to the activation in cortical areas such as anterior cingulate cortex, temporal lobes, right temporal-parietal junction, left lateral precuneus, insula, and cerebellum. Furthermore, positive correlations were presented between the age at onset and activation in the cerebellar anterior lobe, insula, motor cortex, and temporal lobe. These findings all together show that clinical characteristics of the patients, such as the onset, disease severity, and genetic features reflect different brain activities at different stages of the disease.

Several hypotheses have been put forward to explain the brain activity findings in patients with FRDA. Firstly, several studies support the hypothesis of cortico-cerebellar loop damage. This could be due to a disruption of the cerebral–cerebellar circuitry resulting from selective neuronal damage in areas of primary pathology (e.g., DN), followed by secondary functional diaschisis and structural transneuronal degeneration. In the long term, this could lead to the re-organization of the cortico-cerebellar, cortico-striatal, and parietal-frontal loops, and perhaps to the deafferentation of motor and sensory cortices in FRDA. Spinocerebellar tract degeneration and DN atrophy precede cerebellar cortex degeneration, reduced DN activation, and subsequent cerebello–cerebral dysconnectivity and cerebral dysfunction. Though, there is histopathological evidence of primary pathology in the cerebral cortex–specifically in primary motor regions.

A second hypothesis proposes that compensatory mechanisms in FRDA brains emerge to increase the functioning of the remaining intact circuitry and to recruit alternative neural circuits in response to the progressive degeneration and functional failures in areas of primary pathology. This hypothesis can explain the hyperactivations observed in FRDA.

However, the coexistence of atrophy, different disease states between participants at the time of MRI scanning, and small study cohorts make it difficult to interpret decreased or increased BOLD findings that show non-linear activation profiles across individuals at different regions. So far, these findings have pointed to the involvement of areas in the cerebral cortex involved in motor tasks, including the primary, supplementary, and premotor, alongside somatosensory areas. Further investigation is needed to understand the functional changes associated with sensory perception given that ataxia symptoms in FRDA largely result from bottom-up sensory deafferentation, as opposed to top-down motor pathology.

Considering the wide clinical and genetic variability of the patients with FRDA recruited in these studies and also the progressive nature of the disease and likewise of the changes in the brain structure, we believe that future fMRI studies should consider cohort stratification in terms of disease severity, disease duration, and/or GAA triplet ranges. Such an approach would allow for the characterization of the specific functional activation patterns at different disease stages such as pre-symptomatic, immediate onset (or at the diagnosis), a few years after diagnosis when the patient has minimal disability and independent ambulation, after the loss of ambulation but the maintenance of functional independence, and final stages with complete or almost complete need for help. This clinical stratification strategy could be more informative on the time course and location of functional abnormalities, compensatory mechanisms, and changes in cerebellar activation patterns over the different disease stages. Furthermore, a longitudinal follow-up will be required to understand the starting point of the initial cerebral and cerebellar damage. Such an approach may also contribute to solve current uncertainties regarding the timing and/or concurrence of developmental damage and impact of neuronal frataxin deficiency.

### Non-motor (Task) fMRI Studies

The studies designed with non-motor task paradigms represent reasonable attempts to trace *in vivo* cognitive functioning and explain the different patterns of functional activity across cortical and subcortical regions in FRDA. By exploring language production and executive functions, evidence of cerebral cortex areas of hyperactivation and cerebellar areas of hypoactivation emerges as further evidence of the coexistence of neurocognitive deficits and cortico-cerebellar impairments in FRDA (30). Reduced activation in the cerebral cortex, subcortex, and cerebellum, alongside the reduction of cerebral–cerebellar functional connectivity followed by longitudinal activation changes (33), was observed by testing working memory functions (13).

Taken together, fMRI studies of cognitive tasks in FRDA support the conceptualization of system-level dysfunction in this disorder.

Two studies reported RS-fMRI data with no tasks. Cocozza et al. (32) described a picture of lower cerebellar–cerebral and higher cerebro-cerebral functional connectivity in FRDA. A similar connectivity pattern was also observed by Dogan et al. (30) in a task fMRI context. These studies support the compensatory mechanism hypothesis. Significant cortical activity changes were also observed longitudinally within a 1-year period (34). These findings could represent compensation attempts in the cerebellum and other infratentorial structures.

### Cognitive Assessments Findings

Some of the studies included in the review implemented out-of-scanner assessments of cognitive functions (Supplementary Table S1). A more focused and comprehensive review on the FRDA cognitive assessments is necessary to fully address this topic, but here we aim to present a brief overview of the strengths and weaknesses of these 12 studies and provide recommendations for future studies.

A limitation in the interpretation of out-of-scanner neuropsychological findings derives from the fact that cognitive tests, entailing different functions, may explore different indices of cognitive abilities. Consequently, caution should be taken when interpreting and comparing the data.

Motor impairments in FRDA is an important confounding factor in many time-based cognitive tests, which is not always properly accounted for in study design or result interpretation. Conflicting results occur with regard to the cognitive assessment in the FRDA cohorts, but on the other hand, there appears to be consistency in the findings regarding semantic verbal fluency, working memory, visuo-constructive, and visuo-perceptual functions. Shishegar et al. (33) provide the only study with longitudinal assessment of the executive function of patients with FRDA undergoing fMRI. Yet, studies of this kind could benefit from cohort stratification to verify the timing of any compensatory onset with respect to disease progression or other factors. Although social cognition deficits or changes in the personality test results have been reported, attention should be paid to additional key factors such as executive dysfunctions or simply the timing of disease onset and other experiences that can vary between people with FRDA and their healthy peers. All the FRDA cohorts reviewed here are presented as free from psychiatric impairment and yet none of these functions have been formally explored or investigated. We believe that psychiatric assessments should be included, particularly in the early and late stages of the disease. The family and the role of the environment should also be considered when assessing patients with FRDA and analyzing the data.

These considerations point toward the need for common neuropsychological battery of tests used to explore cognitive and neuropsychological functions in FRDA. These tests should consider the age of the patients, including pediatric and adult subjects, and the severity of the disease at the time of testing. In addition, further and specific studies are needed to explore the role of potential confounding factors such as motor impairment, anxiety, depression, and the compensatory mechanisms that influence overall cognitive performance.

### Discussion on Methodology, Technical Aspects, and Paradigms

Most of the studies used high field (3T) or ultra-high field (7T) MR scanners, and half of them were equipped with 32-channel head coils that provide higher signal levels and image homogeneity. Acquisition parameters for EPI sequences varied according to the field strength and technology. In the earlier papers, slice thickness was around 4–5 mm while after 2015 all papers used 3–3.5 mm slice thickness. Regarding temporal resolution, all studies but one (30) used a TR of 2–3 s appearing appropriate for block-design tasks that explore motor functions. Recent advantages in terms of temporal resolution provided by multiband technology have received no investigation yet in FRDA but may allow a more precise investigation of the dynamics of cerebral and cerebellar circuitry.

Despite some minor differences, motor tasks used in these studies consisted of finger tapping activities. This is probably related both to the limitation of the MRI environment and to the impaired and residual motor abilities of FRDA. In Harding et al. (31), task performance was controlled during the in-scan motor task. Such analyses help to understand how the functional activity is modulated by disease severity and residual abilities and should be performed from all the studies.

A point to be considered when dealing with neuroimaging studies is their intrinsic heterogeneity. The number of possible MRI acquisition techniques, sequence set-ups, image processing steps, available software, and measurable features and statistical tests make each study virtually unique due to the impact of each variation in the design and analysis of the final measures (38, 39). Here, we focused on a single technique (fMRI), and the data analysis was performed using either SPM or FSL software (other commonly used fMRI processing suites include AFNI, FreeSurfer, and Braynvoyager). When commenting on the methodological aspects of the studies reviewed here, we have highlighted the common approaches used, providing a starting point for the planning of a study that would be at least partially comparable to the studies included in this review.

#### Open Questions

A series of important questions arise when trying to understand the results of the studies summarized in this review. Is it possible to know which is the first set of neurons impacted in FRDA, and what are the factors of the secondary downstream processes? Subsequently, how and when does this set of principally affected neurons stop neurotransmission to the nearby neurons or to the related circuitry? Is there any difference among the timing and degree of cerebral and cerebellar neuronal damage? Will it be possible to understand whether the neuronal loss occurs in a serial pattern or in parallel circuits? How does frataxin reduction directly affect neuronal activity? Why and how are compensatory mechanisms leading to the hyperactivation of cerebral regions? Can hyperactivation of the cerebellar network be functionally relevant and/or have any implication for treatment?

Answering these questions could eventually open new grounds for circuitry preservation and treatment perspectives. Frataxin-replacement therapies may serve to preserve neural circuitry and/or promote brain function in large-scale brain systems, but meanwhile compensatory mechanisms, once characterized, might be preserved and potentiated with a rehabilitative approach. The primary sites of pathology in FRDA are the dorsal root ganglia and the spinal cord, yet as stated before, no study thus far applied fMRI to these areas. fMRI is now feasible also on spinal cord (40), and interesting information could emerge from the studies conducted on this structure.

Not all the cerebellar sections have low activity, and the degeneration is not homogeneous in all tissues, due to intrinsic difference in the cerebellar cell types. Within a chronic neurodegenerative picture, the glial tissues *per se* are involved and concurrent in the progressive damage (41). The cerebellar cortex and cerebellar nuclei are importantly interconnected to the completion of the internal model of the motor system (42). The intrinsic plasticity of the Purkinje cells and longterm potentiation of the parallel fibers and Purkinje cells are important for the adaptation of locomotion patterns in the healthy brains (43, 44). Despite the current knowledge in the anatomical map of cerebellar damage reported in FRDA (28, 29), further research should be done to complete the picture of the cerebellar circuitry. Cerebellar neuroplasticity is a concept that has been associated to the cerebellar learning, due to a combination of learning, prediction, and timing. In this area, the systematic exploitation of the potentiality of fMRI could provide an important piece contributing to a clearer understanding of the pathophysiology of ataxia.

## Limitations and Future Study Design Recommendations

We did not perform a meta-analysis, and this was due to the limited number of studies and the difficulties in evaluating the results derived from the studies with different protocol designs. The heterogeneity of the design of the fMRI studies did not allow to clearly distinguish between the effect of neural loss and possible compensatory mechanisms. The number of the available studies is limited as it is the routine use of fMRI in the clinical management of patients with FRDA. This limit could be due to the lack of an established consensus on the scanning protocols and paradigms, the lack of adjunctive diagnostic or prognostic value, the demanding technical requirements (and cost) to run a complete rigorous fMRI study. Nevertheless, researchers should remember a unique capacity of fMRI to depict as a whole the ongoing integrated functioning of brain areas: a view no other tool so far can offer.

In conclusion, fMRI (with and without in-scanner tasks) can provide insights into the physiopathology of FRDA. More longitudinal functional studies in FRDA cohorts are needed, and the growing body of data exploring the brain functional substrates of motor activity in FRDA provide a useful foundation for the design of such future studies. Perhaps, by providing *in vivo* and dynamic outcome measures, fMRI could inform basic research studies that investigate important and promising therapies such as gene therapies. Regarding the design of future studies, we expect that multiband technology will become the standard for *in vivo* fMRI investigations, allowing an improvement in time resolution and therefore getting a deeper insight into brain areas and neuronal circuit interactions.

## Data Availability Statement

The original contributions presented in the study are included in the article/Supplementary Material, further inquiries can be directed to the corresponding author/s.

## Author Contributions

MV: concept and design. MV, FF, and SP conducted literature review. All authors take responsibility for manuscript writing and review and contributed to the article, and approved the submitted version.

## Funding

The financial support of the Italian Ministry of Health (project RC2021) is gratefully acknowledged.

## Conflict of Interest

The authors declare that the research was conducted in the absence of any commercial or financial relationships that could be construed as a potential conflict of interest.

## Publisher's Note

All claims expressed in this article are solely those of the authors and do not necessarily represent those of their affiliated organizations, or those of the publisher, the editors and the reviewers. Any product that may be evaluated in this article, or claim that may be made by its manufacturer, is not guaranteed or endorsed by the publisher.

## References

[1] Parkinson MH Boesch S Nachbauer W Mariotti C Giunti P. Clinical features of Friedreich's ataxia: classical and atypical phenotypes. J Neurochem. (2013) 126(Suppl. 1):103–17. 10.1111/jnc.12317 23859346

[2] Campuzano V Montermini L Moltò MD Pianese L Cossée M Cavalcanti F . Friedreich's ataxia: autosomal recessive disease caused by an intronic GAA triplet repeat expansion. Science. (1996) 8:1423–7. 10.1126/science.271.5254.1423 8596916

[3] Campuzano V Montermini L Lutz Y Cova L Hindelang C Jiralerspong S . Frataxin is reduced in Friedreich ataxia patients and is associated with mitochondrial membranes. Hum Mol Genet. (1997) 6:1771–80. 10.1093/hmg/6.11.1771 9302253

[4] Harding IH Lynch DR Koeppen AH Pandolfo M. Central nervous system therapeutic targets in friedreich ataxia. Hum Gene Ther. (2020) 31:1226–36. 10.1089/hum.2020.264 33238751 PMC7757690

[5] Della Nave R Ginestroni A Giannelli M Tessa C Salvatore E Salvi F . Brain structural damage in Friedreich's ataxia. J Neurol Neurosurg Psychiatry. (2008) 79:82–5. 10.1136/jnnp.2007.124297 17634216

[6] França MC Jr D'Abreu A Yasuda CL Bonadia LC Santos da Silva M Nucci A . A combined voxel-based morphometry and 1H-MRS study in patients with Friedreich's ataxia. J Neurol. (2009) 256:1114–20. 10.1007/s00415-009-5079-5 19280106

[7] Selvadurai LP Harding IH Corben LA Stagnitti MR Storey E Egan GF . Cerebral and cerebellar grey matter atrophy in Friedreich ataxia: the IMAGE-FRDA study. J Neurol. (2016) 263:2215–23. 10.1007/s00415-016-8252-7 27522354

[8] Dogan I Romanzetti S Didszun C Mirzazade S Timmann D Saft C . Structural characteristics of the central nervous system in Friedreich ataxia: an *in vivo* spinal cord and brain MRI study. J Neurol Neurosurg Psychiatry. (2019) 90:615–7. 10.1136/jnnp-2018-318422 29945881

[9] Harding IH Chopra S Arrigoni F Boesch S Brunetti A Cocozza S . Brain structure and degeneration staging in friedreich ataxia: magnetic resonance imaging volumetrics from the ENIGMA-ataxia working group. Ann Neurol. (2021) 90:570–83. 10.1002/ana.26200 34435700 PMC9292360

[10] Harding IH Raniga P Delatycki MB Stagnitti MR Corben LA Storey E . Tissue atrophy and elevated iron concentration in the extrapyramidal motor system in Friedreich ataxia: the IMAGE-FRDA study. J Neurol Neurosurg Psychiatry. (2016) 87:1261–3. 10.1136/jnnp-2015-312665 27010617

[11] Solbach K Kraff O Minnerop M Beck A Schöls L Gizewski ER . Cerebellar pathology in Friedreich's ataxia: atrophied dentate nuclei with normal iron content. Neuroimage Clin. (2014) 6:93–9. 10.1016/j.nicl.2014.08.018 25379420 PMC4215469

[12] Stefanescu MR Dohnalek M Maderwald S Thürling M Minnerop M Beck A . Structural and functional MRI abnormalities of cerebellar cortex and nuclei in SCA3, SCA6 and Friedreich's ataxia. Brain. (2015) 138:1182–97. 10.1093/brain/awv064 25818870 PMC5963415

[13] Harding IH Corben LA Storey E Egan GF Stagnitti MR Poudel GR . Fronto-cerebellar dysfunction and dysconnectivity underlying cognition in friedreich ataxia: the IMAGE-FRDA study. Hum Brain Mapp. (2016) 37:338–50. 10.1002/hbm.23034 26502936 PMC6867314

[14] Lindig T Bender B Kumar VJ Hauser TK Grodd W Brendel B . Pattern of cerebellar atrophy in Friedreich's ataxia-using the SUIT template. Cerebellum. (2019) 18:435–47. 10.1007/s12311-019-1008-z 30771164

[15] Fortuna F Barboni P Liguori R Valentino ML Savini G Gellera C . Visual system involvement in patients with Friedreich's ataxia. Brain. (2009) 132:116–23. 10.1093/brain/awn269 18931386

[16] Pagani E Ginestroni A Della Nave R Agosta F Salvi F De Michele G . Assessment of brain white matter fiber bundle atrophy in patients with Friedreich ataxia. Radiology. (2010) 255:882–9. 10.1148/radiol.10091742 20501725

[17] Rizzo G Tonon C Valentino ML Manners D Fortuna F Gellera C . Brain diffusion-weighted imaging in Friedreich's ataxia. Mov Disord. (2011) 26:705–12. 10.1002/mds.23518 21370259

[18] Vieira Karuta SC Raskin S de Carvalho Neto A Gasparetto EL Doring T Teive HA. Diffusion tensor imaging and tract-based spatial statistics analysis in Friedreich's ataxia patients. Parkinsonism Relat Disord. (2015) 21:504–8. 10.1016/j.parkreldis.2015.02.021 25801908

[19] Rezende TJ Silva CB Yassuda CL Campos BM D'Abreu A Cendes F . Longitudinal magnetic resonance imaging study shows progressive pyramidal and callosal damage in Friedreich's ataxia. Mov Disord. (2016) 31:70–8. 10.1002/mds.26436 26688047

[20] Vavla M Arrigoni F Nordio A De Luca A Pizzighello S Petacchi E . Functional and Structural Brain Damage in Friedreich's Ataxia. Front Neurol. (2018) 9:747. 10.3389/fneur.2018.00747 30237783 PMC6135889

[21] Selvadurai LP Harding IH Corben LA Georgiou-Karistianis N. Cerebral abnormalities in Friedreich ataxia: a review. Neurosci Biobehav Rev. (2018) 84:394–406. 10.1016/j.neubiorev.2017.08.006 28823857

[22] Öz G Harding IH Krahe J Reetz K. MR imaging and spectroscopy in degenerative ataxias: toward multimodal, multisite, multistage monitoring of neurodegeneration. Curr Opin Neurol. (2020) 33:451–61. 10.1097/WCO.0000000000000834 32657886 PMC7722013

[23] Selvadurai LP Corben LA Delatycki MB Storey E Egan GF Georgiou-Karistianis N . Multiple mechanisms underpin cerebral and cerebellar white matter deficits in Friedreich ataxia: the IMAGE-FRDA study. IH Hum Brain Mapp. (2020) 41:1920–33. 10.1002/hbm.24921 31904895 PMC7267947

[24] Straub S Mangesius S Emmerich J Indelicato E Nachbauer W Degenhardt KS . Toward quantitative neuroimaging biomarkers for Friedreich's ataxia at 7 Tesla: susceptibility mapping, diffusion imaging, R(2) and R(1) relaxometry. J Neurosci Res. (2020) 98:2219–31. 10.1002/jnr.24701 32731306 PMC7590084

[25] McInnes MDF Moher D Thombs BD McGrath TA Bossuyt PM Clifford T . Preferred reporting items for a systematic review and meta-analysis of diagnostic test accuracy studies: the PRISMA-DTA statement. JAMA. (2018) 319:388–. 10.1001/jama.2017.19163 29362800

[26] Mantovan MC Martinuzzi A Squarzanti F Bolla A Silvestri I Liessi G . Exploring mental status in Friedreich's ataxia: a combined neuropsychological, behavioral and neuroimaging study. Eur J Neurol. (2006) 13:827–35. 10.1111/j.1468-1331.2006.01363.x 16879292

[27] Ginestroni A Diciotti S Cecchi P Pesaresi I Tessa C Giannelli M . Neurodegeneration in friedreich's ataxia is associated with a mixed activation pattern of the brain. A fMRI study. Hum Brain Mapp. (2012) 33:1780–91. 10.1002/hbm.21319 21674694 PMC6870007

[28] Akhlaghi H Corben L Georgiou-Karistianis N Bradshaw J Delatycki MB Storey E . A functional MRI study of motor dysfunction in Friedreich's ataxia. Brain Res. (2012) 1471:138–54. 10.1016/j.brainres.2012.06.035 22771856

[29] Georgiou-Karistianis N Akhlaghi H Corben LA Delatycki MB Storey E Bradshaw JL . Decreased functional brain activation in Friedreich ataxia using the Simon effect task. Brain Cogn. (2012) 79:200–8. 10.1016/j.bandc.2012.02.011 22542844

[30] Dogan I Tinnemann E Romanzetti S Mirzazade S Costa AS Werner CJ . Cognition in Friedreich's ataxia: a behavioral and multimodal imaging study. Ann Clin Transl Neurol. (2016) 3:572–87. 10.1002/acn3.315 27606341 PMC4999591

[31] Harding IH Corben LA Delatycki MB Stagnitti MR Storey E Egan GF . Cerebral compensation during motor function in Friedreich ataxia: the IMAGE-FRDA study. Mov Disord. (2017) 32:1221–9. 10.1002/mds.27023 28556242

[32] Cocozza S Costabile T Tedeschi E Abate F Russo C Liguori A . Cognitive and functional connectivity alterations in Friedreich's ataxia. Ann Clin Transl Neurol. (2018) 5:677–86. 10.1002/acn3.555 29928651 PMC5989773

[33] Shishegar R Harding IH Corben LA Delatycki MB Storey E Egan GF . Longitudinal increases in cerebral brain activation during working memory performance in Friedreich ataxia: 24-month data from IMAGE-FRDA. Cerebellum. (2020) 19:182–91. 10.1007/s12311-019-01094-6 31898277

[34] Vavla M Arrigoni F Toschi N Peruzzo D D'Angelo MG Gandossini S . Sensitivity of neuroimaging indicators in monitoring the effects of interferon gamma treatment in Friedreich's ataxia. Front Neurosci. (2020) 14:872. 10.3389/fnins.2020.00872 33162876 PMC7583645

[35] Vavla M D'Angelo MG Arrigoni F Toschi N Peruzzo D Gandossini S . Safety and efficacy of interferon γ in friedreich's ataxia. Mov Disord. (2020) 35:370–1. 10.1002/mds.27979 31930551

[36] Harding AE. Classification of the hereditary ataxias and paraplegias. Lancet. (1983) 1:1151–5. 10.1016/S0140-6736(83)92879-9 6133167

[37] Woolrich MW Jbabdi S Patenaude B Chappell M Makni S Behrens T . Bayesian analysis of neuroimaging data in FSL. Neuroimage. (2009) 45:S173–86. 10.1016/j.neuroimage.2008.10.055 19059349

[38] Klein A Andersson J Ardekani BA Ashburner J Avants B Chiang MC . Evaluation of 14 nonlinear deformation algorithms applied to human brain MRI registration. Neuroimage. (2009) 46:786–802. 10.1016/j.neuroimage.2008.12.037 19195496 PMC2747506

[39] Bowring A Maumet C Nichols TE. Exploring the impact of analysis software on task fMRI results. Human Brain Mapp. (2019) 40:3362–84. 10.1002/hbm.24603 31050106 PMC6618324

[40] Martin AR Aleksanderek I Cohen-Adad J Tarmohamed Z Tetreault L Smith N . Translating state-of-the-art spinal cord MRI techniques to clinical use: a systematic review of clinical studies utilizing DTI, MT, MWF, MRS, and fMRI. Neuroimage Clin. (2015) 10:192–238. 10.1016/j.nicl.2015.11.019 26862478 PMC4708075

[41] Suzumura A. Neuron-microglia interaction in neuroinflammation. Curr Protein Pept Sci. (2013) 14:16–20. 10.1007/978-1-4614-8313-7 23544747

[42] Wolpert DM Ghahramani Z Jordan MI. An internal model for sensorimotor integration. Science. (1995) 269:1880–2. 10.1126/science.7569931 7569931

[43] Schonewille M Gao Z Boele HJ Vinueza Veloz MF Amerika WE Simek AAM . Reevaluating the role of LTD in cerebellar motor learning. Neuron. (2011) 14:43–50. 10.1016/j.neuron.2011.02.044 21482355 PMC3104468

[44] Vinueza Veloz MF Zhou K Bosman LWJ Potters JW Negrello M Seepers RM . Cerebellar control of gait and interlimb coordination. Brain Struct Funct. (2015) 220:3513–36. 10.1007/s00429-014-0870-1 25139623 PMC4575700

