# Supplementary material for: Functional MRI Studies in Friedreich's Ataxia: A Systematic Review
Source: Front Neurol. 2022 Mar 10;12:802496. doi: 10.3389/fneur.2021.802496 (PMC8960250; doi:10.3389/fneur.2021.802496)
Supplement: Supplementary file 1 [file Data_Sheet_1.pdf]

S.1 Motor task fMRI studies

Mantovan et al. (2006) is the first fMRI study conducted in FRDA with a finger-tapping task. The authors registered in the HCs group activation of the contralateral sensorimotor (SM) cortex (pre-central and post-central gyri) and supplementary motor cortex area (SMA), superior parietal lobule - and inferior parietal lobule. The pattern of activation in the FRDA patients appeared heterogeneous, with very low signal intensity; one patient showed activation in SMA (inferior frontal gyrus, pre-central and post-central gyrus); in three patients the activations were identified in the posterior parietal regions (supramarginal gyrus); in two patients no activation was detected with cluster analysis. No quantitative between-group comparisons were undertaken.

Ginestroni et al. (2012) applied two different tasks: hand tapping and writing of “8”.

Hand tapping task*:* Intragroup analysis showed, for both HCs and FRDA patients, bilateral activation in primary SM mostly in the left side, SMA, insula and in the cerebellar hemispheres (lobules V, VI, VIIIA/B bilaterally).

Intergroup analysis showed that the FRDA cohort had areas of significantly lower activation in the left primary sensorimotor cortex, SMA bilaterally and right middle frontal gyrus, with additional clusters of reduced activation in the left thalamus (nucleus ventralis anterior and nucleus ventralis lateralis) and in the cerebellar hemispheres (lobules right V/VI, at the border zone between left V/VI and in the right side of vermis V).

Writing of the “8” task**:** Intragroup analysis showed, for both HCs and FRDA patients, activation in the left primary SM cortex, right premotor frontal and parietal cortex, right globus pallidus (GP) and putamen, and in the cerebellar hemispheres and vermis (greater activation involving the right lobules V, VI, Crus I, VIIIA/B; left cerebellar hemisphere activation of the lobules V, VI, VIIIA/B and right side of the vermis IV-V).

Intergroup analysis showed in FRDA vs HCs areas of significantly lower activation in the left primary SM and right cerebellar hemisphere (lobule VI, right Crus I). FRDA showed clusters of significantly higher activation in the right precentral and parietal cortex and in the right globus pallidus and putamen.

The cerebral activation in FRDA (right globus pallidus and putamen, anterior cingulum, and parietal cortex) correlated with clinical severity (IACRS score).

Akhlaghi et al. (2012) performed an fMRI study with four different finger tapping tasks: visually cued regular and irregular single finger tapping tasks, a self-paced regular finger tapping task, and a visually cued multi-finger tapping task.

Single finger tapping: Intragroup analysis showed, for both HCs and FRDA, significant activation of the motor-related network including the left primary motor cortex, bilaterally pre-central cortex, bilateral SMA, left somatosensory cortex, left putamen, anterior cerebellum (lobules right V and bilaterally VI), posterior cerebellum (lobule right VIIIA, right crus I) during the regular single finger tapping task. Bilateral activation of the inferior parietal lobule, right middle and inferior frontal gyri and left insula were observed in both groups. Singularly, the FRDA group demonstrated activated areas such as bilateral occipital cortices, left supra-marginal gyrus, right superior temporal gyrus and cerebellum (left crus I and lobule VIIIa). Conversely, the HCs group showed activation in the lateral posterior and ventral posterior thalamic nuclei and left middle frontal gyrus.
Intergroup analysis showed in the FRDA vs HCs group a lower activation in the right cerebellar hemisphere (lobules V, VI), bilateral SMA and right inferior parietal lobule. On the other hand, the FRDA group vs HCs showed higher activation in the cerebellum (lobule left VI, right crus I), left dorsal premotor cortex, left somatosensory cortex and inferior parietal lobule.
***Irregular single finger tapping task*:** Intergroup analysis showed lower activation in the FRDA group in the right cerebellar hemisphere (lobules V, VI), right middle temporal gyrus, right inferior parietal lobule and bilateral SMA. Conversely, the FRDA group showed higher activation in the crus I of the cerebellum bilaterally, left S, bilateral somatosensory cortex, left inferior parietal lobule, right superior parietal lobule, left superior and middle temporal gyrus.
Regular multi-finger tapping task: Intergroup analysis showed lower activation in the FRDA group in middle frontal gyri bilaterally, bilateral somatosensory cortices, bilateral inferior parietal lobule, left premotor cortex and SMA, right superior frontal gyrus and cerebellar hemisphere (lobules V, VI). Conversely, the FRDA group showed higher activation of the left cerebellar hemisphere (lobules V, VI, VIII) and bilateral crus I, left inferior parietal lobule (in a distinct cluster from the HCs), left M1, left middle occipital gyrus and right somatosensory cortex.
***Self-paced finger tapping task*:** Intergroup analysis showed increased activation in the FRDA group vs HCs in right crus I/II, and lower in FRDA vs HCs activation in the left superior frontal gyrus, left central opercular cortex, left somatosensory cortex, left putamen, cerebellar hemispheres (lobules right V, VI bilaterally), bilateral premotor cortex, bilateral inferior parietal lobule and left insula.
Stefanescu et al. (2015) performed opening and closing of right fist during fMRI as part of a multimodal (VBM, SWI, fMRI) study at ultra-high field (7T) and reported a comparative study on three different cohorts of degenerative cerebellar ataxias.
Cerebral activation: Intragroup analysis showed the highest cerebral activation in the left M1 (Brodmann area 4) in both HCs and FRDA, with additional areas of activation derived from the whole brain analysis, such as secondary motor areas, primary and secondary sensory areas.

Intergroup analysis showed higher activation in the left Brodmann area 6 in the FRDA.

Cerebellar cortex activation: Hand movements led to strong activations within the cerebellar cortex (lobules V, VI, VIII, greater on the right side) in the HCs that corresponds to the motor somatotopic hand representation in the cerebellum. These hand movement areas are less activated in the FRDA group vs the HCs group.

Intergroup analysis showed significantly lower activation in the FRDA group vs the HCs, with prominent activation in the lobules V and VI.

Dentate nuclei (DN): Intragroup analysis showed in HCs activation related to hand movements in the DN bilaterally, with strongest activation in the right dorsorostral DN and extended into the dorsocaudal and ventral parts of the DN. Intragroup analysis in the FRDA group observed no significant activations. Intergroup analysis showed significantly lower DN activation in the FRDA group vs HCs.

No significant correlations with the clinical severity were registered in the FRDA group.

Harding et al. (2017) performed a self-paced, regularly timed, single-finger tapping paradigm and a visually cued, irregularly timed, multi-finger tapping paradigm.

During self-paced, single-finger tapping: Intragroup analysis in both groups showed the activation of the left M1, somatosensory cortices, midline SMA, right inferior parietal lobule, and right cerebellar lobule V. The HCs group in addition showed significant activations in the right and left ventral premotor cortex, leftinsula, and right cerebellar lobule VIIIB. Intergroup analysis showed in FRDA significantly reduced activations in right cerebellum lobule VI. Greater disease severity correlated with lesser activation in the SMA, left anterior insula, left inferior parietal lobule, and left superior temporal gyrus. A median-split analysis indicated that individuals with lower disease severity (mean FARS = 58) had greater activations in these regions relative to controls, while the FRDA subcohort with high disease severity (mean FARS = 95) had lesser activations relative to controls.

Visually cued, multifinger task: Intragroup analysis showed activation in both groups in areas of motor systems M1, somatosensory, SMA, dorsal and ventral premotor, intraparietal sulci, inferior and superior parietal lobules, caudate nuclei, and cerebellar lobules V to VII and VIIb to VIIIb. The FRDA group in addition showed activations in the dorsolateral prefrontal cortex bilaterally, the dorsal precuneus, and posterior aspects of the right middle temporal gyrus.

Intergroup analysis showed that FRDA elicited greater activations in bilateral dorsolateral prefrontal cortex, ventrolateral premotor areas, intraparietal sulci, caudate nuclei, medial precuneus, right fusiform gyrus and leftcerebellar crus I.

The GAA1 size showed an inverse correlation with less task-related activation in the anterior cingulate cortex, right temporal-parietal junction and right lateral precuneus.
Vavla et al. (2018) performed a multi finger-tapping task bimanually.
R hand motor task (contrast right side greater than left side): Intragroup analysis in both groups evidenced activation of the left M1, L insula, and R superior cerebellar hemisphere (lobules V, VII, VIII). Intergroup analysis registered no differences during movement of the dominant hand (right).
L hand motor task (contrast L>R): Intragroup analysis evidenced activations in the right M1 cortex, right insula, and left superior cerebellar hemisphere (lobules V, VI, VIII) in both groups. Intergroup analysis registered weaker activation in the left superior cerebellar hemisphere in the FRDA group vs HCs during the movement of the non-dominant hand (left).
Correlations between functional activations and clinical indicators were significant as follows: positive correlations occurred between age at onset and activation in the cerebellar anterior lobe, insula, motor cortex, and temporal lobe; negative correlations between disease severity measures and GAA1 size relative to fMRI activation in the cerebellum, insula, and temporal lobes.
Vavla et al. (2020b) performed a motor task to monitor the effect of IFNγ treatment with a multi finger-tapping task bimanually (see above) applied at four time-points: T-6 (prior to the baseline), T0 (baseline, i.e., beginning of a 6 months treatment), T6 (end of the 6 months treatment) and T12 months (6 months of washout period).
Considering the different time-points of the study: an increased activation was registered between the T-6 and T6 in the left M1 during the movement of the dominant hand (right), between the T0 and T6 in the left M1 during the movement of the non-dominant hand (left), and between the T6 and T12 in the left inferior cerebellum during the movement of non-dominant hand.
Inverse correlations were found between the disease severity index and the functional activity in the right M1 for both hands at T0 and T6.

S2 Non-Motor task fMRI studies

Georgiou-Karistianis et al. (2012) performed the Simon effect task (incongruent minus congruent stimuli) in order to examine FRDA-related differences in cognitive activation patterns; the Simon task relies on selective attention and response inhibition.
Intragroup analysis in the HCs group: the response to both congruent and incongruent stimuli showed the activation of an extensive range of bilateral cortical and sub-cortical regions (superior,

middle and inferior prefrontal cortices, SMA, premotor, M1 and somatosensory cortex, anterior and posterior portion of the cingulate, insula, superior parietal cortex , inferior parietal lobule, occipital cortex, thalamic nuclei, caudate nuclei, putamen, cerebellar lobules VI and VII/VIII, crus I and II, vermis VI). The response to the Simon effect (incongruent minus congruent) led to significant activations in the following regions bilaterally: the superior, middle and inferior prefrontal cortices, the posterior portion of the cingulate, insulae, thalamic nuclei, caudate nuclei, superior and inferior parietal lobule and the cerebellum (lobules V, VI, crus I/II).

Intragroup analysis in the FRDA group: the response to *congruent stimuli* showed bilateral activations in the superior and middle prefrontal cortices, SMA, premotor (PMA),M1 and somatosensory cortex, insulae, superior and inferior parietal cortices, occipital cortex, thalamic nuclei, caudate nuclei, putamen, cerebellar lobules VI and VII/VIII, vermis VI). The response to *incongruent stimuli* overlapped with the congruent stimuli areas distribution. The Simon effect (incongruent minus congruent) triggered bilateral significant activations in the superior and middle prefrontal cortices, insulae, superior and inferior parietal lobule, occipital cortices, thalamic nuclei, caudate nuclei and the cerebellar right lobule VI.

Intergroup analysis for the Simon effect *(incongruent minus congruent)*: the FRDA group showed a significantly lower bilateral activation relative to FRDA in regions of the superior and middle prefrontal cortices, superior temporal gyri, lateral occipital cortices, caudate nuclei, L inferior prefrontal cortex, right inferior parietal lobule, left insulae and left cerebellar lobule V and VI.

No significant correlations were found between the clinical measures and the BOLD response in various ROIs explored.

Functional connectivity for the Simon effect: In the HCs group, a significant correlation was observed between cortical (dorsolateral prefrontal cortex)-subcortical regions (caudate and putamen), cortical (dorsolateral prefrontal cortex)-thalamus, and sub-cortical (putamen)-thalamus. In the FRDA group, significant correlations were demonstrated bilaterally between the cerebellum-M1, M1- dorsolateral prefrontal cortex (right hemisphere), M1-subcortical regions (caudate) (left hemisphere). The FRDA group showed a reduced number of significant correlations relative to HCs between cortical-subcortical regions.

Dogan et al. (2016) used a verbal fluency task that relied on phonological and semantic fluency, and overt and covert speech.

Verbal fluency task in fMRI: Intergroup analysis showed higher activity in the FRDA cohort in the left BA44, L anterior insula, left precentral gyrus, premotor cortex, SMA, anterior mid-cingulate cortex and middle occipital gyrus. Similarly, lower functional activity in the ventral posterior cingulate cortex was observed in FRDA patients. ***Overt versus covert speech contrast:*** Intergroup

analysis showed that the FRDA group vs HCs had a reduced activity in the left anterior insula, right cerebellar lobule VI, Crus I bilaterally (greater on the right side), and also a higher activity in the left precentral and middle frontal gyrus, right inferior parietal lobe and bilateral occipital regions.

Semantic fluency task in fMRI: Intergroup analysis showed reduced activity in FRDA vs HCs in the anterior cerebellum (lobules I–IV). Similarly, for ***overt versus covert speech***, the FRDA group vs HCs group showed increased activity in M1.

Verbal fluency task in ROI-to-ROI functional connectivity: Intergroup analysis showed in FRDA vs HCs a reduced connectivity in the right cerebellar lobule VI, L BA44/insula.

In the FRDA group, there were anticorrelations for the connectivity between right lobule VI and left insula relative to impaired phonemic fluency performance and higher positive functional connectivity between cortical ROI (BA44- M1/ACC, BA45-M1).

Semantic fluency in ROI-to-ROI functional connectivity: Intergroup analysis observed in the FRDA group vs HCs a pattern of higher functional co-activation between cortical ROIs (BA45-ACC, BA45-Insula) and between cerebellar ROIs (lobules VIIB-Crus I, VIIB-Crus II). **For the overt speech,** they found similar results in BA44-M1, VIIB-Crus II.

Harding et al. (2016) applied an n-back working memory paradigm useful to activate cerebellar-cerebral cognitive networks and to provide information regarding executive, maintenance and attention processes.

Cerebellar fMRI Activations: intragroup analysis in the HCs group evidenced robust activations (two-back- >0-back) bilaterally across superior cerebellar cortex (lobule VI, crus I), and inferior cerebellar cortex (lobule VIIB). Intragroup analysis in the FRDA group registered a significant activation in the above-mentioned areas but with reduced spatial extent. Both groups showed similar bilateral activations in the dorsal and lateral regions of the DN.

Intergroup analysis revealed significant reduction of the activation in the FRDA group vs HCs in the cerebellar cortex (lobule VI bilaterally, VIIB). No evidence of intergroup differences in the DN was registered.

Cerebral fMRI Activations: Intragroup analysis showed significant task-related activations in both groups bilaterally in the rostrolateral prefrontal cortices (rlPFC), anterior insulae, and medial prefrontal cortices encompassing areas of the dorsal anterior cingulate cortices and pre-SMA; and deactivations in response to task bilaterally in the middle/posterior insulae, and middle/posterior cingulate cortices and ventral-anterior thalamus.

Intergroup analysis showed a lower activation in the FRDA group vs HCs in the left rlPFC, anterior insula and ventrolateral thalamus, and deactivation in the middle insula and parietal operculum.

Cerebellar-Cerebral Connectivity: Intragroup analysis of the task-invariant connectivity between a seed (cerebellar right lobule VI) and selected regions of the cerebral cortex in the HCs group were robust throughout the insular, rostrolateral prefrontal and cingulate cortices bilaterally. Intragroup analysis in the FRDA group showed comparable effects only to the bilateral insular regions. Intergroup analysis showed in the FRDA group a reduction in the connectivity bilaterally in the rlPFC and both anterior and middle cingulate cortices. Interestingly, the HCs group showed a greater decrease in the task-related connectivity relative to FRDA between the cerebellar cortex and bilateral rlPFC, left anterior insula and anterior cingulate cortex.

Correlations were observed between the disease severity and both the magnitude of functional activations in the lobule VI bilaterally and the task-related activation in the right DN. No correlations were evident between disease severity and the activation magnitude in the cerebrum or in cerebellar-cerebral connectivity.

Cocozza et al. (2018) is a seed-based RS-fMRI study that investigated functional connectivity in FRDA relative to HCs.

Intergroup analysis showed significantly increased connectivity in the FRDA group relative to HCs in the following connections: bilateral paracingulate gyrus-right middle frontal gyrus; right superior frontal gyrus-bilateral angular gyrus; and left middle temporal gyrus-cingulate gyrus. Decreased connectivity in FRDA vs HCs was found between right middle frontal gyrus-cerebellar lobule VI.

No correlations emerged between the clusters with impaired functional connectivity and the neuropsychological and clinical indexes.

Shishegar et al. (2020) performed a longitudinal fMRI study with a working memory task.

Intragroup analysis in the FRDA group showed significant task-related activations at both T0 and T24m in the SMA bilaterally, left insula and inferior frontal gyrus (pars triangularis, pars opercularis); no significant activations in the cerebellar cortex; and over time, significantly increased activation in the right inferior frontal gyrus and insula. Intragroup analysis in the HCs group evidenced significant task-related activations at both T0 and T24m in the bilateral insula, inferior frontal gyrus, SMA and bilaterally in the cerebellar cortex (lobule VI, crus I); no significant intragroup activation changes were observed over time.

Intergroup analysis showed in the FRDA vs HCs group a greater increase over time of functional activation in bilateral insula and inferior frontal gyrus.

Significant correlations emerged at the follow up timepoint (T24m) between right insula and inferior frontal gyrus and the disease severity index (negative correlation), and to the disease onset (positive correlation). No significant correlations were found between the disease severity and the

functional activation in cerebral areas that showed longitudinal changes; similarly, no changes occurred in the neurocognitive measures. In both groups, no significant correlations were observed between the longitudinal changes in the neurocognitive measures and changes in brain activation over time.

Vavla et al. (2020b) performed a RS-fMRI acquisition to study the activity of several networks (default mode network, hippocampus, bilateral fronto-parietal, bilateral fronto-temporal, visual and motor) using independents components analysis (ICA).

The analysis between the T0 and T12 time-points showed significantly modified connectivity within the default mode network (DMN), sensorimotor network and the L fronto-parietal networks.
