# Supplementary material for: Functional MRI Studies in Friedreich's Ataxia: A Systematic Review
Source: Front Neurol. 2022 Mar 10;12:802496. doi: 10.3389/fneur.2021.802496 (PMC8960250; doi:10.3389/fneur.2021.802496)
Supplement: Supplementary file 2 [file Table_1.pdf]

S1 Table 1 Psychological and cognitive investigation, findings and correlations

Reference	Cognitive, Psychological measures	Cognition/Psy findings
**Mantovan et al., 2006**	Cognitive assessment, Language, Memory, Calculations, RT for Finger tapping, Attention/Information Processing/Planning, Implicit Learning Task, Personality	Normal cognitive assessment. Impaired: mean verbal fluency, short-term verbal memory, visual memory, slower at movement programming, poor learning capacity. Personality: increased irritability, poor impulsive control, blunting of affect, poor self-portraying.
**Ginestroni et al., 2012**	none	n.a.
**Akhalaghi et al., 2012**	BDI: FRDA 7±7.9, HC 3.7±4.7	No differences in BDI intergroup.
**Georgiou-Karistianis et al., 2012**	BDI: 7±7.9 FRDA vs 3.7±4.7 HC, NART cognitive assessment score 117.1±3.1 FRDA vs 121.5±2.6 HC	Pre-morbid cognitive assessment in FRDA. Greater Simon effect difference (incongruent minus congruent) in FRDA vs HC.
**Stefanescu et al., 2015**	none	n.a.
**Dogan et al., 2016**	HADS: FRDA 10.2 ± 6.28, HC 6.27 ± 4.71	Impaired phonemic and semantic verbal fluency, working memory, attention and social cognition. Social cognition deficit.
**Harding et al., 2016**	BDI, N-Back Task (RT magnitude, RT variance, accuracy), NART	HC: Task performance and rlPFC activations in cerebral cortex correlated for SD and RT, anterior insula for SD and trending to significance for RT, more variable and/or poorer task performance. Task-related cerebello-insular dynamic connectivity in L hemisphere correlated to behavioral variance in HC only.
**Harding et al., 2017**	Motor performance measures. Offline motor behavior speeded motor performance and paced motor precision	n.a.
**Cocozza et al., 2018**	Language, cognitive assessment, Executive, memory, visuoperception/visuospatial functions.	Poor performance on MOCA. No language impairment. Except verbal working memory and categorization, FRDA performed worse than HC in tests assessing executive and attentive abilities.
**Vavla et al., 2018**	cognitive assessment tests	Cognitive assessment in FRDA normal in 13 (out of 19), IDD in 2, bordeline in 4. Non harmonic cognitive assessment profile.
**Shishegar et al., 2020**	Digit span forward/backward tests, Hayling's sentence completion test, Stroop, TMTA/B.	Executive functions (Stroop): FRDA worse than HC, Attentive functions: FRDA worse at f-up.
**Vavla et al., 2020**	none	n.a.
