# Supplementary material for: Functional MRI Studies in Friedreich's Ataxia: A Systematic Review
Source: Front Neurol. 2022 Mar 10;12:802496. doi: 10.3389/fneur.2021.802496 (PMC8960250; doi:10.3389/fneur.2021.802496)
Supplement: Supplementary file 3 [file Table_2.pdf]

S2 Table Data for Figure 2.

	Both	HC	FRDA	HC vs FRDA
1 Frontal L	5	5	4	HC: 2 ; FRDA: 1;
1 Frontal R	4	2	2	HC: 2; FRDA: 2;
1 Insula L	3	1		HC: 1;
1 Insula R	2			
1 Occipital L			1	FRDA: 1;
1 Occipital R			1	
1 Parietal L	2	1	2	FRDA: 2 ; HC:1;
1 Parietal R	3		1	FRDA: 3 ; HC: 1;
1 Caudate L	1			FRDA: 1;
1 Caudate R	1			FRDA:1;
1 Putamen L	1			HC: 1;
1 Putamen R	1		2	FRDA: 1;
1 Pallidum L				
1 Pallidum R	1		1	FRDA: 1;
1 Thalamus L		2		HC: 1;
1 Thalamus R				
1 Temporal L				FRDA: 1;
1 Temporal R			3	HC: 1 ; FRDA: 1;
0 Cerebellum Crus1 L	1		2	FRDA: 1;
0 Cerebellum Crus1 R	1	1		HC: 1; FRDA: 2;
0 Cerebellum Crus2 L				
0 Cerebellum Crus2 R				FRDA: 1;
0 Cerebellum 3 L				HC: 1;
0 Cerebellum 3 R				
0 Cerebellum 4 5 L	3			FRDA: 1; HC: 1;
0 Cerebellum 4 5 R	5	2		HC: 3;
0 Cerebellum 6 L	4			HC:2 ; FRDA: 1;
0 Cerebellum 6 R	4	2		HC: 4;
0 Cerebellum 7b L	1	1	1	HC: 1;
0 Cerebellum 7b R	2			
0 Cerebellum 8 L	3	1	2	FRDA: 1; HC: 1;
0 Cerebellum 8 R	5	2		HC: 1;
0 Cerebellum 9 L		1	1	
0 Cerebellum 9 R				
0 Cerebellum 10 L			1	
0 Vermis 4 5	1	1		HC: 1;

Column HC vs FRDA: FRDA values means number of papers that report higher activation in FRDA than HC; HC values means number of papers thta report higher activation in HC than in FRDA. Number stands for number of papers that reported the data/area.

Number stands for number of papers that reported the data/area.
HC: means HC higher than FRDA and FRDA lower than HC
FRDA: means FRDA higher than HC and HC lower than FRDA
